# The Prevalence, Progress and Risk Factor Control of Chronic Kidney Disease in Chinese Adults With Type 2 Diabetes Mellitus in Primary Care

**DOI:** 10.3389/fendo.2022.859266

**Published:** 2022-06-10

**Authors:** Lingwang An, Qiuzhi Yu, Hong Tang, Xianglan Li, Dandan Wang, Qi Tang, Haiyang Xing, Yali He, Xiaona Zhao, Shuhui Zhao, Yaujiunn Lee, Juming Lu

**Affiliations:** ^1^ Department of Endocrinology, Beijing Ruijing Diabetes Hospital, Beijing, China; ^2^ Department of Endocrinology, Heilongjiang Ruijing Diabetes Hospital, Haerbin, China; ^3^ Department of Share-care center, Chengdu Ruien Diabetes Hospital, Chengdu, China; ^4^ Department of Endocrinology, Taiyuan Diabetes Hospital, Taiyuan, China; ^5^ Department of Endocrinology, Lanzhou Ruijing Diabetes Hospital, Lanzhou, China; ^6^ Department of Metabolism and Endocrinology, Lee’s Clinic, Pingtung, Taiwan; ^7^ Department of Endocrinology, The General Hospital of the People’s Liberation Army, Beijing, China

**Keywords:** chronic kidney disease (CKD), diabetes, type 2, CKD progress, primary care

## Abstract

**Objective:**

This study aimed to evaluate the prevalence of chronic kidney disease (CKD) in Chinese adults with T2DM in primary care, and the association of HbA_1c_, blood pressure (BP) and triglycerides (TG), i.e. ABC control at follow up (FU) with the progress and regression of CKD.

**Methods:**

A total of 5123 patients with ≥3 measurements of estimated glomerular filtration rate (eGFR), urinary albumin-to-creatinine ratio (UACR), HbA_1c_, BP, LDL-C and TG, and FU ≥ 12 months were included into final analysis. The presence of CKD was defined as the presence of albuminuria (UACR ≥ 30 mg/g), impaired eGFR (eGFR < 60 ml/min/1.73 m^2^) or both, and was categorised as low, moderate and high/very high risk. The change of CKD risk for outcome was categorised as stable (no change), progress (risk increase) and regress (risk decrease) from baseline to the last visits (LV).

**Results:**

The prevalence of CKD, impaired eGFR and albuminuria was 29.6%, 5.8% and 27.1% at baseline, with 70.4%, 20.3%, 7.0% and 2.3% of patients distributed in low, moderate, high and very high risk group. There were 3457 (67.5%), 1120 (21.8%) and 546 (10.7%) patients had CKD outcome risk stable, progressed and regressed respectively. The proportion of patients reaching targets of BP ≤ 130/80 mmHg, HbA_1c_<7.5%, LDL-C<2.60 mmol/L increased from baseline to FU and LV, together with increased usage of insulin, RAS inhibitors and lipid lowering medications. After multivariable adjustment, the HbA_1c_<7.5% (OR: 0.66, 95%CI 0.56-0.78), TG< 1.7 mmol/L (OR: 0.81, 95%CI 0.68-0.96) at FU and BP ≤ 130/80 mmHg at LV (OR: 0.82, 95%CI 0.70-0.95) was negatively associated with CKD outcome risk progress.

**Conclusion:**

The prevalence of CKD was high with 21.8% of patients progressing to higher CKD outcome risk at FU, attention should be paid on long term and better ABC control.

## Introduction

Diabetic kidney disease (DKD) is one of the most common complications of diabetes (1), characterised by albuminuria, reduced glomerular filtration rate (GFR) or both (2). The estimated GFR (eGFR) was used to determine kidney function stage and was calculated based on serum concentration of creatinine, cystatin C or both using given formulas (3–6). A decline in eGFR is represented as a one-directional process, and once initiated, it progresses to end-stage kidney disease (ESKD), albeit at widely differing individual rates; the abnormal urinary albumin excretion can regress, stay the same or progress (3). The global percentage of prevalent ESKD patients with diabetes increased from 19.0% in 2000 to 29.7% in 2015, with the most rapid increase rates and highest average rates (44.1%) observed in the Western Pacific Region (7). According to some recent years’ reports, diabetes was the third cause (27.1%) for CKD onset, the leading cause (40.5%) for CKD progression and the most common cause (45%) for ESKD (8, 9). In the general population, the overall prevalence of chronic kidney disease (CKD) ranged from 9.9% to 16.8% in the Zhejiang and Henan provinces, and the prevalence of DKD was less than 5% (10–12). In patients with type 2 diabetes mellitus (T2DM), the prevalence of CKD increased greatly and ranged from 27.1% to 52.3% in different Chinese studies (10, 13–17).

To prevent the onset and progress of CKD in diabetes, the modifiable risk factors, including tobacco and alcohol use, physical activity, stress, body mass index (BMI), haemoglobin A1C (HbA_1c_) level, blood pressure (BP), blood lipids, GFR and albuminuria, need to be controlled (18). The lower prevalence of albuminuria was observed over time (from 20.8% in 1988–1994 to 15.9% in 2009–2014) among United States adults with diabetes, and was attributable to the lowering of mean blood glucose, blood pressure and lipid levels with a higher rate of prescribed glucose and lipid lowering medications and renin-angiotensin-aldosterone system (RAAS) inhibitors (19).The mean decrease of HbA_1c_(from 8.1% to 7.6%), systolic BP (SBP, from136.3 to 130.1 mmHg), diastolic BP (DBP, from76.2 to 68.9 mmHg), low-density lipoprotein cholesterol (LDL-C, from3.55 to 2.66 mmol/L) and triglycerides (TG, from2.25 to 1.61 mmol/L) was considerable; while the usage of RAAS inhibitors (from 24.4% to 56.2%) and lipid lowering medications (from 17.0% to 51.8%) increased significantly (19).The HbA_1c_, BP and LDL-C/TG control (i.e. ABC control) in Chinese T2DM patients with CKD were still poor in recent years reports, with a mean HbA_1c_ of 8.3–9.2%, SBP ≥ 135 mmHg, DBP ≥ 80 mmHg, LDL-C ≥ 3.1 mmol/L and TG ≥ 2.2 mmol/L (13, 15, 17). Given the projected increase in the number of people diagnosed with diabetes and complicated with CKD, poor ABC control will induce a significant increase in the number of people with ESKD over the next few decades and will impose a heavy burden on our healthcare system.

More data from different regions are still needed to understand the prevalence and the metabolic control of CKD in Chinese patients with T2DM. This study aimed to evaluate the prevalence of CKD in Chinese adults with T2DM in primary care, and the association of ABC control at follow up (FU) with the progress and regression of CKD. The results would provide us important epidemiological information and be useful for understanding the present situation and long-term direction for diabetes care.

## Materials and Methods

### Study Design

This was a retrospective multicentre cross-sectional study based on medical records included in the Diabetes Share-care Information System (DSIS) of the Ruijing Diabetes Chain Hospitals (RDCH). The RDCH comprises five primary care medical institutes located in Beijing, Taiyuan, Chengdu, Harbin and Lanzhou. The DSIS was developed for diabetic patients’ registration, follow-up and preservation of clinical information, biochemical measurements and medications at entry and during follow-up. With oral consent, patients would be registered on the DSIS and would become involved in comprehensive risk factor assessments and screening for diabetic complications. Patients would also get multidisciplinary management from healthcare professionals, including doctors in different major, nurses and dietitians. The RDCH has implemented the DSIS since 2016.

This analysis was based on the DSIS data at baseline and FU. Collected variables included sociodemographic status (age, gender, education), disease history (hypertension, cardiovascular disease and diabetic complications), lifestyle (smoking, drinking, exercise), BMI, anthropometric measurements (height, weight, resting blood pressure), biochemical parameters (serum creatinine [SCr], fasting plasma glucose [FPG], HbA_1c_, LDL-C, TG, high-density lipoprotein cholesterol [HDL-C], total cholesterol [TC]). This study was approved by the ethics committee of the Beijing Ruijing Diabetes Hospital and The Declaration of Helsinki was followed. Due to the nature of this study (i.e. a retrospective database), patient consent was not required.

### Inclusion Criteria

1. Patients with T2DM; ≥ 18 years; age at diabetes diagnosis ≥18 years old
2. Patients had ≥3records of eGFR, urinary albumin-to-creatinine ratio (UACR), HbA_1c_, LDL-C, TG and BMI measurements and FU ≥ 6 months

### Exclusion Criteria

1. Patients with an age at diabetes diagnosis <18 years old, or with a fasting plasma C peptide <100 pmol/L
2. Patients with a history of malignant tumour, blindness, or serious thyroid disease
3. Patients with an FPG <3.9 mmol/L or >33.0 mmol/L
4. Patients with a BMI <18.5 kg/m^2^
5. Patients with urinary tract infection
6. Patients with measurements of eGFR, UACR, HbA_1c_, LDL-C and TG ≤ 2 and FU ≤ 5 months
7. Patients with baseline eGFR< 14.9 mL/min/1.73m^2^.

### Measurements and Categories

HbA_1c_ measurements were tested with high-performance liquid chromatography using the HA-8180 (ARKAY, Inc., Kyoto, Japan) and MQ-2000PT (Medconn Diagnostics, Shanghai, China) analysers. Biochemical parameters, including SCr, LDL-C, UACR, were tested using the TBA-120FR (Toshiba, Beijing, China), CS-1200 (DIRUI, Changchun, China) and BS-450 (Mindray, Shenzhen, China) automatic biochemical analysers. UACR was measured using spot urine samples collected at a random time of the day. All the biochemical measurements were in regular quality control and met the local internal quality control standards.

The following simplified Chinese Modification of Diet in Renal Disease (MDRD) equation was used to calculate eGFR: MDRD = 175 × serum creatinine (mg/dL) ^− 1.234^ × age (years) ^− 0.179^ × 0.79 (if female) (5). The GFR categories (G1: eGFR ≥ 90.0 mL/min/1.73m^2^; G2: eGFR 60.0–89.9 mL/min/1.73m^2^; G3a: eGFR 45.0–59.9 mL/min/1.73m^2^; G3b: eGFR 30.0–44.9 mL/min/1.73m^2^; G4: eGFR 15.0–29.9 mL/min/1.73m^2^; G5: eGFR< 14.9 mL/min/1.73m^2^) and UACR categories (normal albuminuria [A1]: UACR < 30.0 mg/g; microalbuminuria [A2]: UACR 30.0–300.0 mg/g; macroalbuminuria [A3]: UACR > 300.0 mg/g) were classified according to the relative guidelines (20, 21). The presence of CKD was defined as the presence of albuminuria (UACR ≥ 30 mg/g), impaired eGFR (eGFR < 60 ml/min/1.73 m^2^) or both, and was categorised as low, moderate and high/very high risk (2, 22, 23). The change of CKD risk for outcome was categorised as stable (no change), progress (risk increase) and regress (risk decrease) from baseline to the last visit (LV) or the 12^th^ visit if FU measurements≥12.

The BMI was categorised as normal: 18.5–24.0 kg/m^2^, overweight: BMI 24.0–27.9 kg/m^2^ and obesity: BMI ≥ 28.0 kg/m^2^. Targets of HbA_1c_, BP, LDL-C and TG was defined as <7.5%, ≤130/80 mmHg, <2.6 mmol/L, and <1.7 mmol/L respectively (20, 21).The mean values of HbA_1c_, LDL-C and TG were analysed as the average of all available values from the second visit to the LV or the 12^th^ visit if FU measurements≥12.

Participants with a history of myocardial infarction, coronary revascularization, heart failure, or stroke, transient ischaemic attack or cerebral haemorrhage were considered to have a history of cardiovascular disease (CVD). Reporting of hypertension, dyslipidaemia, peripheral arterial disease (PAD) and retinopathy were based on both disease history and medical records of detection. The duration of diabetes was defined as the difference between a participant’s age at examination and age when diagnosed with diabetes. Treatment for hyperglycaemia was categorised as non-insulin or insulin (insulin only or insulin plus other treatment) usage. The use of sodium-glucose cotransporter-2 inhibitors (SGLT2i), glucagon-like peptide-1 receptor agonist (GLP-1RA), and angiotensin-converting enzyme inhibitors (ACEI) or angiotensin receptor blockers (ARB) was further categorised.

### Outcomes

The primary outcome demonstrated was the prevalence of CKD in Chinese adults with T2D in primary care hospitals at baseline and LV. The secondary outcome demonstrated was the progression and regression of CKD outcome risk from baseline to LV. The association of ABC control at baseline, FU and LV with the progression and regression of CKD risk were also demonstrated.

### Statistical Analysis

Categorical variables were expressed as numbers (%). Continuous variables were expressed as mean ± standard deviation (SD) for normally distributed variables, and median (25% and 75% quartile) for variables not normally distributed. Differences in patient characteristics, ABC control and medications among categories of risk for CKD outcomes (low, moderate and high/very high risk) were studied using the chi-square test for categorical variables and a one-way analysis of variance (ANOVA) or Kruskal–Wallis test for continuous variables where appropriate. Posthoc analysis were checked with Bonferroni. Binary logistic regression was used to estimate the odds ratio (OR) for the association of ABC control at baseline and FU with the progression and regression of CKD outcome risk with multivariable adjustments. A two-sided *P* value of <0.05 was considered to be statistically significant. All analyses were performed using the SPSS 22.0 software.

## Results

### Study Population

**Figure 1:**
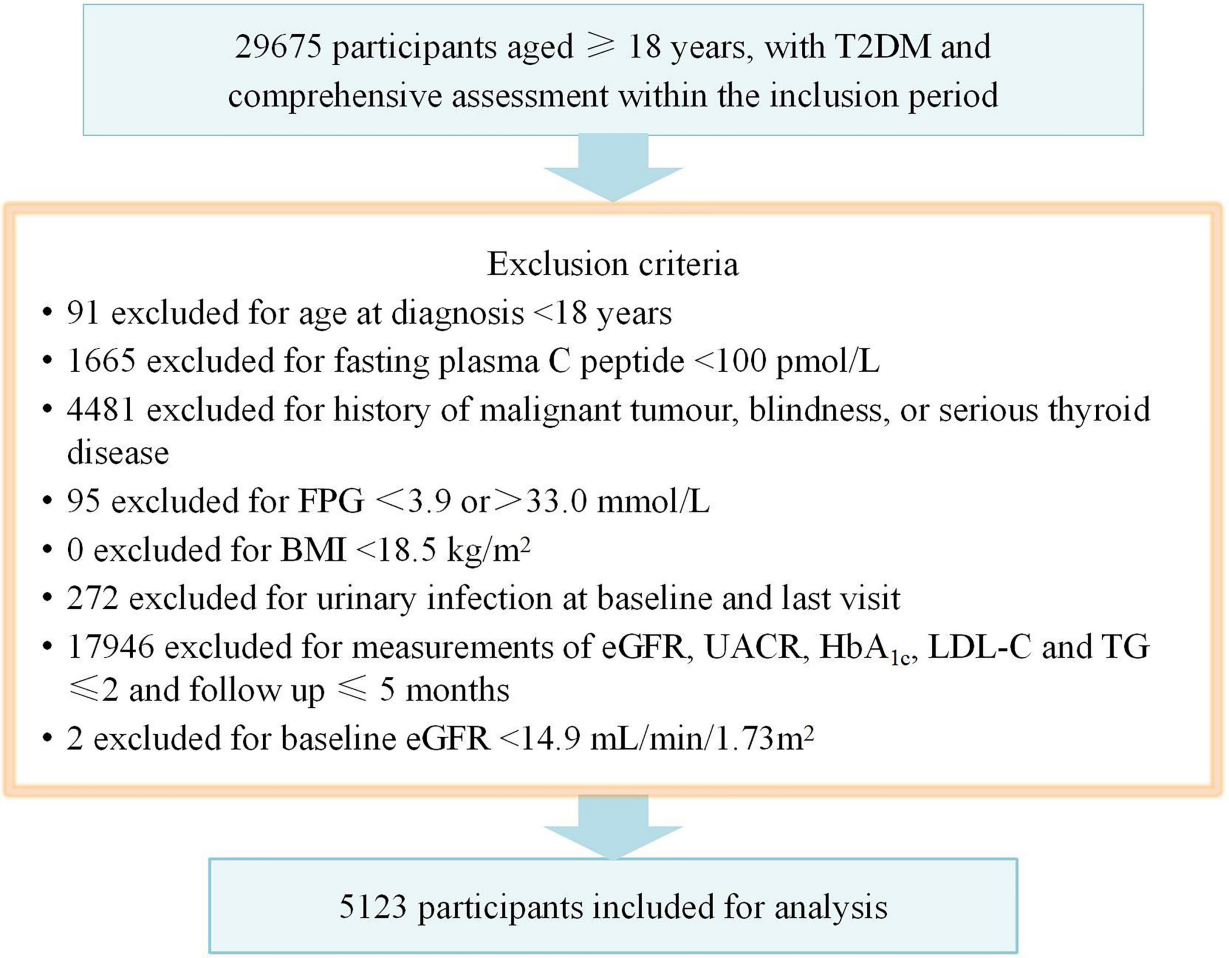
Flow chart showing the exclusion criteria. T2DM, type 2 diabetes mellitus; BMI, body mass index; FPG, fasting plasma glucose.

During the inclusion period lasting from January 29, 2016 to November 6, 2021, there were 5213 patients were included into final analysis, as shown in **Figure 1**.

The demographic and clinical characteristics of all individuals included in the study are shown in **Table 1**. The median age of our patients was 61.3 years old with male accounts for 57.3% of patients. The median duration of diabetes was 8.7 years. The proportion of patients having hypertension, dyslipidaemia, CVD, PAD and diabetic retinopathy were 45.8%, 42.1%, 25.1%, 24.1% and 18.9% respectively.

**Table 1:** Characteristics of the overall study population.

Characteristics	All (n=5123)
Age (years)	61.3 (54.3, 67.3)
Age at diagnosis (years)	52.3 (45.4, 59.3)
Gender (male)	2937 (57.3)
Diabetes duration (years)	8.7 (4.6, 13.3)
Follow up (months)	30 (21, 38)
Education	
≤junior middle school	2737 (54.3)
≥senior high school	2304 (45.7)
Place of visit	
Outpatient department	2784 (55.4)
Inpatient department	2245 (44.6)
Current smoker	803 (15.7)
Current drinker	831 (16.2)
Regular exercise	2674 (52.5)
**Comorbidities/complications**	
Hypertension	2344 (45.8)
Dyslipidemia	2158 (42.1)
CVD	1288 (25.1)
Peripheral arterial disease	1233 (24.1)
Retinopathy	969 (18.9)
Neuropathy	2766 (54.0)

CVD, Cardiovascular disease (angina, acute myocardial infarction, percutaneous coronary stent implantation and bypass, heart failure) and cerebrovascular diseases (stroke/transient ischemic attack).

### The Prevalence, Progress and Regress of CKD

**Figure 2:**
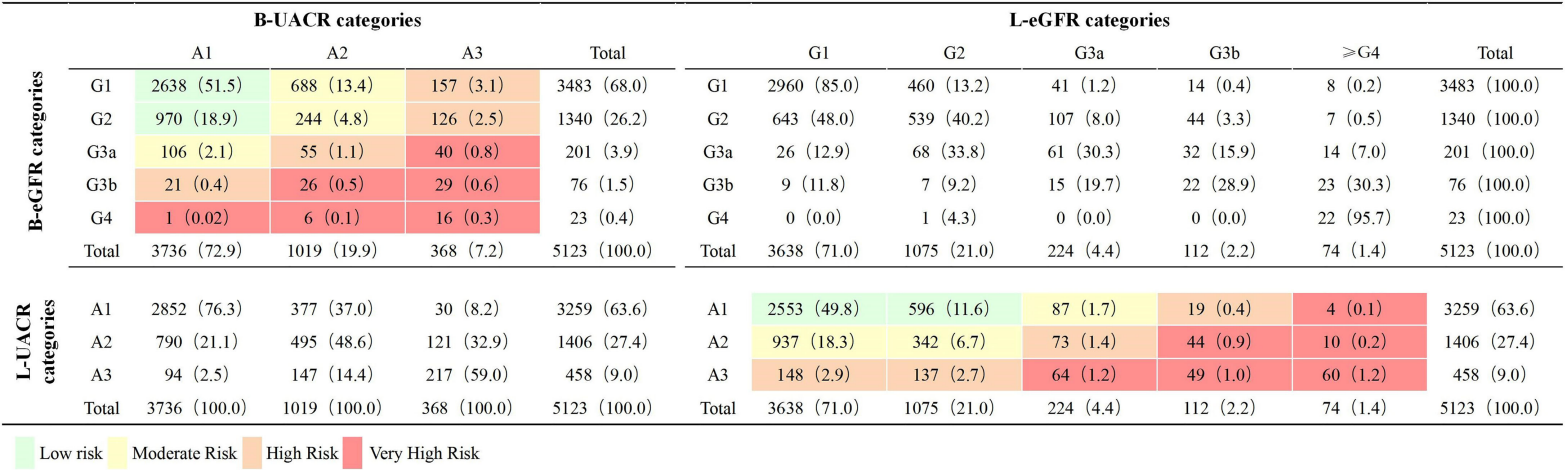
Distribution by eGFR and UACR categories at baseline and Last visit (n = 5123). Classification is based on KDIGO 2012 Clinical Practice Guidelines for the Evaluation and Management of Chronic Kidney Disease (4). eGFR, estimated glomerular filtration rate (mL/min/1.73m^2^); UACR, urinary albumin-to-creatinine ratio (mg/g); B, baseline; L, last visit. G1, eGFR ≥ 90.0 mL/min/1.73m^2^; G2, eGFR 60.0–89.9 mL/min/1.73m^2^; G3a: eGFR 45.0–59.9 mL/min/1.73m^2^; G3b: eGFR 30.0–44.9 mL/min/1.73m^2^; G4: eGFR 15.0–29.9 mL/min/1.73m^2^; A1: UACR < 30.0 mg/g; A2: UACR 30.0–300.0 mg/g; A3: UACR > 300.0 mg/g.

The prevalence of CKD, impaired eGFR (with or without albuminuria) and albuminuria (with or without impaired eGFR) was 29.6%, 5.8% and 27.1% at baseline and 38.6%, 8.0% and 36.4% at last visit. The distribution of low, moderate, high and very high risk for the CKD outcome was 70.4%, 20.3%, 7.0% and 2.3% at baseline, and 61.5%, 26.6%, 7.4% and 4.4% at last visit. There were 3457 (67.5%), 1120 (21.8%) and 546 (10.7%) patients had CKD outcome risk stable, progressed and regressed respectively. Both progress and regression of UACR and eGFR category was found from baseline to last visit. The regression of UACR was found in 37.0% and 41.0% of patients at stage A2 and A3. The progress of UACR was found in 23.6% and 14.4% of patients at stage A1 and A2 (**Figure 2**).

### The ABC Control and Medications at Baseline and FU by Risk Categories

The ABC control and medications at baseline and FU for patients by CKD risk categories are shown in **Table 2**. Patients in moderate or high/very high risk group were mainly older, male, obese, had a longer duration of diabetes, higher baseline BMI, SBP, DBP, HbA_1c_, LDL-C and TG level, higher proportion of patients treated with insulin and ACEI/ARB. The proportion of patients reaching targets of BP ≤ 130/80 mmHg, HbA_1c_<7.5%, LDL-C<2.60 mmol/L increased from baseline to FU and LV, together with increased usage of insulin, RAS inhibitors and lipid lowering medications. However, patients reaching targets of BP ≤ 130/80 mmHg, HbA_1c_<7.5% or TG<1.70 mmol/L at FU were still fewer in moderate or high/very high risk group compared to low risk group.

**Table 2:** Characteristics of study population by risk categories for CKD outcome.

Characteristics	Total (n=5123)	Low risk (n=3608)	Moderate risk (n=1038)	High/very high risk (n=477)	P
Age (years)	61.3 (54.3, 67.3)	60.8 (53.9, 66.6)	62.1 (55.0, 69.0)***	63.3 (56.5, 71.3)***^##^	<0.001
Gender (male)	2937 (57.3)	1989 (55.1)	638 (61.5)***	310 (65.0)***	<0.001
Diabetes duration (years)	8.7 (4.6, 13.3)	8.3 (4.3, 12.6)	8.8 (4.8, 14.3)**	10.9 (5.8, 18.4)***^###^	<0.001
Body mass index (kg/m^2^)	25.2 ± 3.1	25.0 ± 3.0	25.5 ± 3.3***	25.6 ± 3.4***	<0.001
**Measurements at B and FU**					
B-SBP (mmHg)	130.5 ± 15.2	128.7 ± 14.2	133.1 ± 15.6***	138.8 ± 17.9***^###^	<0.001
L-SBP (mmHg)	127.1 ± 10.7	126.5 ± 10.1	127.9 ± 11.5**	130.1 ± 12.6***^##^	<0.001
B-DBP (mmHg)	77.4 ± 9.6	76.7 ± 9.2	79.1 ± 10.0***	79.8 ± 10.9***	<0.001
L-DBP (mmHg)	75.5 ± 7.6	75.2 ± 7.4	76.2 ± 7.7***	76.5 ± 8.3**	<0.001
B-BP ≤ 130/80	2733 (53.3)	2089 (57.9)	480 (46.2)***	164 (34.4)***^###^	<0.001
L-BP ≤ 130/80	3355 (65.7)	2451 (68.2)	645 (62.3)***	259 (54.8)***^##^	<0.001
B-HbA_1c_ (%)	7.6 (6.6, 9.0)	7.4 (6.5, 8.7)	8.0 (6.9, 9.7)***	8.5 (7.1, 9.9)***^#^	<0.001
M-HbA_1c_ (%)	7.4 (6.7, 8.4)	7.3 (6.6, 8.3)	7.6 (6.8, 8.6)***	7.8 (6.9, 8.9)***^#^	<0.001
L-HbA_1c_ (%)	7.4 (6.6, 8.6)	7.3 (6.6, 8.5)	7.5 (6.7, 8.6)**	7.6 (6.8, 8.9)***	<0.001
B-HbA_1c_<7.5	2373 (46.3)	1831 (50.7)	384 (37.0)***	158 (33.1)***	<0.001
M-HbA_1c_<7.5	2643 (51.6)	1963 (54.4)	481 (46.3)***	199 (41.7)***	<0.001
L-HbA_1c_<7.5	2568 (50.9)	1887 (52.9)	480 (47.2)**	201 (43.5)***	<0.001
B-LDL-C (mmo/L)	2.77 ± 0.94	2.74 ± 0.91	2.82 ± 0.99*	2.88 ± 1.05**	0.001
M-LDL-C (mmo/L)	2.66 ± 0.75	2.65 ± 0.73	2.69 ± 0.80	2.72 ± 0.86	0.089
L-LDL-C (mmo/L)	2.64 ± 0.90	2.63 ± 0.86	2.67 ± 0.98	2.70 ± 1.01	0.145
B-LDL-C<2.60	2236 (43.6)	1614 (44.7)	437 (42.1)	185 (38.8)*	0.026
M-LDL-C<2.60	2435 (47.5)	1736 (48.1)	480 (46.2)	219 (45.9)	0.430
L-LDL-C<2.60	2534 (50.2)	1807 (50.7)	503 (49.4)	224 (48.4)	0.544
B-TG (mmo/L)	1.63 (1.15, 2.44)	1.57 (1.12, 2.31)	1.80 (1.22, 2.69)***	1.83 (1.29, 2.75)***	<0.001
M-TG (mmo/L)	1.69 (1.22, 2.44)	1.63 (1.18, 2.36)	1.78 (1.32, 2.64)***	1.89 (1.35, 2.77)***	<0.001
L-TG (mmo/L)	1.60 (1.13, 2.41)	1.56 (1.10, 2.30)	1.68 (1.17, 2.61)***	1.82 (1.26, 2.80)***^#^	<0.001
B-TG<1.70	2677 (52.3)	1998 (55.4)	467 (45.0)***	212 (44.4)***	<0.001
M-TG<1.70	2577 (50.3)	1907 (52.9)	467 (45.0)***	203 (42.6)***	<0.001
L-TG<1.70	2711 (53.5)	1978 (55.3)	520 (50.7)**	213 (45.9)***	<0.001
**Medications at B**					
Insulin	2657 (51.9)	1753 (48.6)	563 (54.2)**	341 (71.5)***^###^	<0.001
ACEI/ARB	1362 (26.6)	838 (23.2)	318 (30.6)***	206 (43.2)***^###^	<0.001
SGLT2i	133 (2.6)	78 (2.2)	44 (4.2)***	11 (2.3)	0.001
Lipid lowing	1708 (33.3)	1216 (33.7)	339 (32.7)	153 (32.1)	0.679
Statins	1573 (30.7)	1122 (31.1)	311 (30.0)	140 (29.4)	0.624
GLP1-RA	55 (1.1)	32 (0.9)	17 (1.6)*	6 (1.3)	0.108
**Medications at FU**					
Insulin	3133 (61.2)	2082 (57.7)	666 (64.2)***	385 (80.7)***^###^	<0.001
ACEI/ARB	2075 (40.5)	1257 (34.8)	519 (50.0)***	299 (62.7)***^###^	<0.001
SGLT2i	885 (17.3)	556 (15.4)	246 (23.7)***	83 (17.4) ^##^	<0.001
Lipid lowing	2624 (51.2)	1803 (50.0)	555 (53.5)*	266 (55.8)*	0.016
Statins	2447 (47.8)	1686 (46.7)	513 (49.4)	248 (52.0)*	0.047
GLP1-RA	192 (3.7)	124 (3.4)	50 (4.8)*	18 (3.8)	0.119

CKD, Chronic kidney disease; B, baseline; M, mean of all the follow up measurements; L, last or 12^th^ measurement; BMI, body mass index; SBP, systolic blood pressure; DBP, diastolic blood pressure; LDL-C, low density lipoprotein cholesterol; TG,triglycerides; ACEI, angiotensin converting enzyme inhibitors; ARB, angiotensin receptor antagonist; SGLT2i, sodium-glucose cotransporter-2 inhibitors; GLP1-RA, Glucagon-like peptide-1 receptor agonists.

Moderate or High/very high vs. Low risk,* P<0.05, ** P<0.01, ***P<0.001; Moderate vs High/very high,^#^ P<0.05,^##^ P<0.01, ^###^ P<0.001. Categorical variables were expressed as numbers (%). Continuous variables were expressed as mean ± SD for normally distributed variables, and as median (25% and 75% quartile) for non-normally distributed variables.

### The ABC Control at Baseline and FU by CKD Outcome Risk

The ABC control at baseline and FU for patients by CKD outcome risk are shown in **Table 3**. Compared with patients in stable group, patients in regression group were mainly obese, having HbA_1c_ measurements>3 per year, having higher baseline but similar mean and LV HbA_1c_ and TG level as well similar proportion of patients reaching targets of BP ≤ 130/80 mmHg, HbA_1c_<7.5%orTG<1.70 mmol/L at FU and LV; patients in progress group were mainly older, having longer duration of diabetes, and fewer proportion of patients having HbA_1c_ measurements>3 per year, reaching targets of BP ≤ 130/80 mmHg, HbA_1c_<7.5% or TG<1.70 mmol/L at baseline, FU and LV.

**Table 3:** Characteristics of study population by CKD outcome.

Characteristics	Total (n=5123)	Stable (n=3457)	Regress (n=546)	Progress (n=1120)	P
Age (years)	61.3 (54.3, 67.3)	60.8 (53.8, 66.6)	60.8 (54.3, 67.3)	63.4 (56.4, 69.6)***^###^	<0.001
Gender (male)	2937 (57.3)	1971 (57.0)	329 (60.3)	637 (56.9)	0.342
Diabetes duration (years)	8.7 (4.6, 13.3)	8.4 (4.4, 13.0)	7.7 (3.7, 13.3)	10.0 (5.6, 14.6)***^###^	<0.001
Follow up (months)	30.0 (21.0, 38.0)	29 (21, 37)	25 (16, 34)***	33 (24, 41)***^###^	<0.001
HbA_1c_ measurements per year					<0.001
<2	1837 (35.9)	1220 (35.3)	169 (31.0)**	448 (40.0)**^###^	
2∼3	1984 (38.7)	1348 (39.0)	203 (37.2)	433 (38.7)	
>3	1302 (25.4)	889 (25.7)	174 (31.9)	239 (21.3)	
**Measurements at B and FU**					
B-Body mass index (kg/m^2^)	25.2 ± 3.1	25.1 ± 3.1	25.5 ± 3.3**	25.4 ± 3.1**	<0.001
Normal	1903 (37.1)	1332 (38.5)	191 (35.0)*	380 (33.9)**	0.002
overweight	2319 (45.3)	1561 (45.2)	239 (43.8)	519 (46.3)	
obesity	901 (17.6)	564 (16.3)	116 (21.2)	221 (19.7)	
B-eGFR categories					<0.001
G1	3483 (68.0)	2495 (72.2)	357 (65.4)***	631 (56.3)***^###^	
G2	1340 (26.2)	812 (23.5)	95 (17.4)	433 (38.7)	
G3a	201 (3.9)	85 (2.5)	67 (12.3)	49 (4.4)	
≥G3b	99 (1.9)	65 (1.9)	27 (4.9)	7 (0.6)	
B-UACR categories					<0.001
A1	3736 (72.9)	2781 (80.4)	46 (8.4)***	909 (81.2)^###^	
A2	1019 (19.9)	488 (14.1)	377 (69.0)	154 (13.8)	
A3	368 (7.2)	188 (5.4)	123 (22.5)	57 (5.1)	
B-BP ≤ 130/80	2733 (53.3)	1931 (55.9)	257 (47.1)***	545 (48.7)***	<0.001
L-BP ≤ 130/80	3355 (65.7)	2326 (67.5)	355 (65.3)	674 (60.4)***	<0.001
B-HbA_1c_ (%)	7.6 (6.6, 9.0)	7.4 (6.5, 8.8)	8.0 (6.9, 9.8)***	7.9 (6.9, 9.3)***	<0.001
M-HbA_1c_ (%)	7.4 (6.7, 8.4)	7.3 (6.6, 8.2)	7.4 (6.7, 8.4)	7.8 (7.0, 8.8)***^###^	<0.001
L-HbA_1c_ (%)	7.4 (6.6, 8.6)	7.3 (6.6, 8.4)	7.2 (6.5, 8.4)	7.8 (6.9, 9.0)***^###^	<0.001
B-HbA_1c_<7.5	2373 (46.3)	1751 (50.7)	199 (36.4)***	423 (37.8)***	<0.001
M-HbA_1c_<7.5	2643 (51.6)	1915 (55.4)	285 (52.2)	443 (39.6)***^###^	<0.001
L-HbA_1c_<7.5	2568 (50.9)	1832 (53.7)	294 (55.1)	442 (40.1)***^###^	<0.001
B-LDL-C (mmo/L)	2.77 ± 0.94	2.77 ± 0.92	2.82 ± 1.00	2.75 ± 0.99	0.294
M-LDL-C (mmo/L)	2.66 ± 0.75	2.67 ± 0.74	2.66 ± 0.76	2.65 ± 0.79	0.734
L-LDL-C (mmo/L)	2.64 ± 0.90	2.65 ± 0.88	2.63 ± 0.91	2.62 ± 0.95	0.656
B-LDL-C<2.60	2236 (43.6)	1504 (43.5)	229 (41.9)	503 (44.9)	0.496
M-LDL-C<2.60	2435 (47.5)	1631 (47.2)	260 (47.6)	544 (48.6)	0.719
L-LDL-C<2.60	2534 (50.2)	1705 (50.0)	278 (51.7)	551 (50.2)	0.760
B-TG (mmo/L)	1.63 (1.15, 2.44)	1.60 (1.12, 2.37)	1.71 (1.20, 2.65)**	1.71 (1.20, 2.60)**	<0.001
M-TG (mmo/L)	1.69 (1.22, 2.44)	1.65 (1.18, 2.39)	1.71 (1.28, 2.44)*	1.82 (1.33, 2.60)***	<0.001
L-TG (mmo/L)	1.60 (1.13, 2.41)	1.55 (1.10, 2.32)	1.60 (1.13, 2.40)	1.75 (1.21, 2.69)***^##^	<0.001
B-TG<1.70	2677 (52.3)	1866 (54.0)	261 (47.8)**	550 (49.1)**	0.002
M-TG<1.70	2577 (50.3)	1807 (52.3)	265 (48.5)	505 (45.1)***	<0.001
L-TG<1.70	2711 (53.5)	1908 (55.7)	289 (53.6)	514 (46.8)***^##^	<0.001

CKD, Chronic kidney disease; B, baseline; M, mean of all the follow up measurements; L, last or 12^th^ measurement; BMI, body mass index; SBP, systolic blood pressure; DBP, diastolic blood pressure; LDL-C, low density lipoprotein cholesterol; TG, triglycerides; ACEI, angiotensin converting enzyme inhibitors; ARB, angiotensin receptor antagonist; SGLT2i, sodium-glucose cotransporter-2 inhibitors; GLP1-RA, Glucagon-like peptide-1 receptor agonists.

Regress/Progress vs. Stable,* P<0.05, ** P<0.01, ***P<0.001; Progress vs Regress, ^##^ P<0.01, ^###^P<0.001. Categorical variables were expressed as numbers (%). Continuous variables were expressed as mean ± SD for normally distributed variables, and as median (25% and 75% quartile) for non-normally distributed variables.

### The ABC Control and the Progress/Regression of CKD Outcome Risk

After multivariable adjustment, the M-HbA_1c_<7.5% and M-TG< 1.7 mmol/L was positively associated with regression of CKD outcome risk with OR of 1.36 (95%CI 1.04-1.79) and 1.39 (95%CI 1.06-1.82) respectively; and negatively associated with progress of CKD outcome risk with OR of 0.66 (95%CI 0.56-0.78) and 0.81 (95%CI 0.68-0.96) respectively in model 1. The L-HbA_1c_<7.5% and L-TG< 1.7 mmol/L was also associated with CKD risk regression and progress in model 2 without inclusion of M-HbA1c<7.5% and M-TG< 1.7 mmol/L. The baseline and LV BP ≤ 130/80mmHg was associated with CKD risk progress in the 2 models (**Table 4**).

**Table 4:** The association of ABC control and CKD outcome.

Characteristic	OR (95%CI) for regression, *P* value		OR (95%CI) for progression, *P* value	
	Model 1	Model 2	Model 1	Model 2
B-BP ≤ 130/80	1.16 (0.91-1.47), 0.236	1.13 (0.88-1.45), 0.333	0.81 (0.70-0.94), 0.006	0.82 (0.71-0.95), 0.009
L-BP ≤ 130/80	1.25 (0.98-1.60), 0.074	1.24 (0.97-1.60), 0.093	0.82 (0.70-0.95), 0.007	0.82 (0.71-0.96), 0.011
B-HbA1c<7.5%	0.81 (0.61-1.07), 0.135	0.78 (0.60-1.02), 0.072	0.71 (0.60-0.84), <0.001	0.65 (0.55-0.76), <0.001
M-HbA1c<7.5%	1.36 (1.04-1.79), 0.024	——	0.66 (0.56-0.78), <0.001	——
L-HbA1c<7.5%	——	1.58 (1.22-2.05), 0.001	——	0.77 (0.65-0.91), 0.002
B-TG<1.70 mmol/L	1.04 (0.79-1.36), 0.802	1.15 (0.88-1.49), 0.305	0.95 (0.80-1.13), 0.574	0.95 (0.81-1.12), 0.531
M-TG<1.70 mmol/L	1.39 (1.06-1.82), 0.018	——	0.81 (0.68-0.96), 0.014	——
L-TG<1.70 mmol/L	——	1.36 (1.05-1.76), 0.021	——	0.75 (0.64-0.88), 0.001

Adjusted for categories of age, gender, diabetes duration, baseline body mass index, eGFR, UACR, education, current smoking, drinking and physical activity status; Model 1: L-HbA1c<7.5% and L-TG<1.70 mmol/L were not included; Model 2: M-HbA1c<7.5% and M-TG<1.70 mmol/L were not included.

## Discussion

The prevalence of CKD in this study (29.6%) was similar to that in a Hong Kong (29.7%) and Jiangsu (31.0%) study, with relatively different prevalence of impaired eGFR (5.8%, 11.6% and 6.5%) and albuminuria (27.1%, 23.6% and 28.9%) (14, 24). The prevalence of albuminuria was often higher than that of reduced eGFR in T2DM patients (13, 14, 19, 24, 25), however, a pretty high prevalence of albuminuria (45.3%) with much lower prevalence of reduced eGFR (6.3%) was also reported in central Chinese urban population with T2DM (12). All the variables associated with CKD risk, including diabetes duration, HbA_1c_, BP and TG level etc, as well different equation used to calculate eGFR, would contribute to the different CKD prevalence (26). The similar CKD prevalence in this study and in Hongkong and Jiangsu study might arise from similar baseline ABC control, with median/mean HbA_1c_ of 7.6%, 7.2%, and 7.56%, SBP of 130, 134 and 129 mmHg, DBP of 77, 74 and 77 mmHg, LDL-C of 2.77, 2.3 and 2.87mmol/L, and TG of 1.63, 1.6 and 1.47 mmol/L (11,39). The prevalence of CKD would increase to around 53% in polyclinics or hospitalized patients with diabetes duration ≥10 years and worse ABC control (3, 17). A relentless focus on improving ABC control would be important for reducing CKD prevalence.

Patients in this study were involved in a chronic shared care model since 2016, receiving comprehensive risk factor assessments, screening for diabetic complications, monitoring ABC control and adjusting treatment regularly. Patients with T2DM enrolled in this kind of chronic, integrated or shared care models were more likely to receive care that was patient centred and collaborative, compared with patients who received routine care regardless of the setting, and would be beneficial to improving ABC control and delaying the complications of diabetes (27–30). Thus we found SBP, DBP, HbA_1c_, LDL-C level decreased at FU, and the proportion of patients reaching targets of BP ≤ 130/80 mmHg, HbA_1c_<7.5%, LDL-C<2.60 mmol/L increased from baseline to FU and LV. The increased usage of insulin, RAS inhibitors, SGLT2i and lipid lowering for low, moderate and high/very high risk group indicated intensified and personalized treatment adjustment. Compared to baseline value, the median/mean HbA_1c_ (7.4%), SBP (127 mmHg), DBP (75 mmHg), LDL-C (2.64 mmol/L) and TG (1.60 mmol/L) at LV was closer to that in Hongkong population with T2DM enrolled in a management programme implemented since 2009, although better in low risk group and worse in high/very high risk group (14). However, an increase in the prevalence of CKD (38.6%), impaired eGFR (8.0%) and albuminuria (36.4%) was still found after 30 (21, 38) months follow up. We wonder if there were any space for improvement in respect of modifiable risk factors discussed here, i.e., ABC control.

The increased CKD prevalence in this study mainly resulted from the increased prevalence of albuminuria and the 21.8% of patients progressing to higher CKD outcome risk. The significant longer diabetes duration (10.0 vs 7.7 years), lower rate of HbA1c <7.5% at FU (39.6% vs 52.2%) and LV (40.1% vs 55.1%), as well TG< 1.7mmol/L at LV (46.8% vs 53.6%) in progress group than in regress group might lead to CKD outcome progress instead of regression. After multivariable adjustment, the mean and last BP ≤ 130/80 mmHg, HbA_1c_<7.5% and TG< 1.7 mmol/L was associated with CKD risk progress in different models, indicating the importance of more aggressive ABC control in clinical practice. In the Action to Control Cardiovascular Risk in Diabetes (ACCORD) trial, 3.7 years of intensive glycemic control targeting HbA_1c_<6.0% reduced the incidence of the composite kidney outcome in the follow-on 7.7 years and primarily driven by a reduction in incident macroalbuminuria, indicating the importance of long-term intensive glycemic control (31). Aiming to a 30% decrease in UACR during a 2-year baseline period would lead slow progression of CKD and 17% risk reduction of ESKD (32). In a study focus on effects of BP on renal outcomes, a follow-up SBP > 130 mmHg was associated with an increase hazard ration (2.33) for renal outcomes with referent to SBP ≤ 130 mmHg (33). Among a subset of ACCORD-BP trial, UACR was 30% lower in the intensive BP control group (SBP<120 mm Hg) at year 2 compared to a less intensive BP target (SBP <140 mm Hg) (34). Thus, more efforts should be paid on improving ABC control at FU although it might be more difficult in daily clinical practice than in prospective intervention study. Compared to United states and Singapore study, the usage of RAAS inhibitors (56.2%, 59.5% vs 40.5%) and lipid lowering medications (51.8%, 81.2% vs 51.2%) in this study might also have space to increase (19, 26).

There were several limitations in this study. First, it was uncertain that the CKD in our subjects was due to diabetic nephropathy, since they might also have abnormal kidney structures or other diseases. However, the term ‘diabetic nephropathy’ has been considered outdated and is avoided in clinical practice with CKD recommendations (19). Second, we used creatinine-based GFR estimates, which have been criticised for lack of accuracy in patients with diabetes, especially within the normal or high range of GFR (6). Nevertheless, creatinine-based eGFR is still widely adopted in current clinical practice. Third, the dose of ACEI/ARB was not recorded and analysed in this study, since titrate to the max tolerated doses or even more aggressive doses would effectively reduce urinary protein excretion rates, and the efficacy would be enhanced in terms of better BP control and better HbA_1c_ control (35, 36). Therapeutic inertia (TI) in initiation or intensification of ACEI/ARB treatment might exist, since a 40.3% prevalence of TI was reported in Hongkong study (37), it probably also existed in our clinical practice and need further investigation.

## Conclusion

In conclusion, this study provided us information about the prevalence of CKD and metabolic risk factors management at baseline and follows up, as well the association of ABC control and CKD outcome risk. Patients with T2DM had intensified and personalized treatment adjustment in this share care management model. Since 21.8% of patients progressing to higher CKD outcome risk, attention should be paid on long term and better ABC control.

## Data Availability Statement

The original contributions presented in the study are included in the article/supplementary material. Further inquiries can be directed to the corresponding authors.

## Ethics Statement

This study was conducted in accordance with the Declaration of Helsinki and approved by the ethics committee of Beijing Ruijing Diabetes Hospital.

## Author Contributions

Conception and design of the work: LA; Data collection: LA, QY, HT, XL, DW, QT, HX, YH, XZ, and SZ Supervision: LA, YL and JL; Analysis and interpretation of the data: LA, YL and JL; Statistical analysis: LA Drafting the manuscript: LA; Critical revision of the manuscript: LA, YL and JL; Approval of the final manuscript: all authors.

## Conflict of Interest

The authors declare that the research was conducted in the absence of any commercial or financial relationships that could be construed as a potential conflict of interest.

## Publisher’s Note

All claims expressed in this article are solely those of the authors and do not necessarily represent those of their affiliated organizations, or those of the publisher, the editors and the reviewers. Any product that may be evaluated in this article, or claim that may be made by its manufacturer, is not guaranteed or endorsed by the publisher.
